# Peri-operative fluid strategy and post-operative acute kidney injury in cardiac surgery patients: any role for pre-operative statin therapy?

**DOI:** 10.1186/s13054-015-1174-4

**Published:** 2015-12-29

**Authors:** Patrick M. Honore, Rita Jacobs, Inne Hendrickx, Elisabeth De Waele, Viola Van Gorp, Herbert D. Spapen

**Affiliations:** ICU Department, Universitair Ziekenhuis Brussel, Vrije Universiteit Brussel, 101 Laarbeeklaan, 1090, Brussels, Belgium

Acute kidney injury (AKI) is a common complication associated with increased morbidity and mortality in cardiac surgery patients. In this context, the recent paper by Kim et al., showing that a peri-operative liberal ‘saline- and starch-based’ fluid management significantly enhanced the incidence of severe post-operative AKI in off-pump cardiac surgery patients, is to be acclaimed [1].

With regard to the potential culprit(s) triggering this AKI, the authors pointed at ‘usual suspects’ such as excess chloride and intrinsic starch-induced renal tubular lesions. However, it is noteworthy that patients in the at-risk group were less likely (*p* = 0.001!) to be receiving statins before surgery [1]. Statins may prevent kidney injury through inhibition of post-operative inflammatory processes. Compared with statin-naive subjects, cardiac surgery patients taking statins indeed had reduced levels of circulating C-reactive protein, tumour necrosis factor alpha, myeloperoxidase, and pro-inflammatory interleukin (IL)-1, IL-6, and IL-8 and higher concentrations of the anti-inflammatory IL-10 [2]. The largest meta-analysis to date, including nearly 60,000 cardiac surgery patients, revealed a 13 % reduction of post-operative AKI in patients pre-operatively treated with statins [3].

Whether statins embrace the kidney as friend or foe is debatable but probably depends upon the type of study cohort (e.g. septic vs. non-septic), the intervention (e.g. surgery), and pre-existing chronic kidney disease. For example, agent-dependent ‘high potency’ statin doses increase the risk of AKI in a general patient population [4] whereas the use of corresponding high doses in cardiac surgery patients appears to exhibit a reno-protective effect [5].

## Abbreviations

AKI: acute kidney injury
IL: interleukin

## References

[1] Kim JY, Joung KW, Kim KM, Kim MJ, Kim JB, Jung SH (2015). Relationship between a perioperative intravenous fluid administration strategy and acute kidney injury following off-pump coronary artery bypass surgery: an observational study. Crit Care.

[2] Garwood S (2010). Statins and cardiac surgery. J Cardiothorac Vasc Anesth.

[3] Wang J, Gu C, Gao M, Yu W, Yu Y (2015). Preoperative statin therapy and renal outcomes after cardiac surgery: a meta-analysis and meta-regression of 59,771 patients. Can J Cardiol.

[4] Dormuth CR, Hemmelgarn BR, Paterson JM, James MT, Teare GF, Raymond CB (2013). Use of high potency statins and rates of admission for acute kidney injury: multicenter, retrospective observational analysis of administrative databases. BMJ.

[5] Mithani S, Kuskowski M, Slinin Y, Ishani A, McFalls E, Adabag S (2011). Dose-dependent effect of statins on the incidence of acute kidney injury after cardiac surgery. Ann Thorac Surg.

[6] Huffmyer JL, Mauermann WJ, Thiele RH, Ma JZ, Nemergut EC (2009). Preoperative statin administration is associated with lower mortality and decreased need for postoperative hemodialysis in patients undergoing coronary artery bypass graft surgery. J Cardiothorac Vasc Anesth.

[7] Virani SS, Nambi V, Polsani VR, Lee VV, Elayda M, Kohsaka S (2010). Preoperative statin therapy decreases risk of postoperative renal insufficiency. Cardiovasc Ther.

[8] Argalious M, Xu M, Sun Z, Smedira N, Koch CG (2010). Preoperative statin therapy is not associated with a reduced incidence of postoperative acute kidney injury after cardiac surgery. Anesth Analg.

[9] Nemati MH, Astaneh B (2015). The effects of preoperative statins on the incidence of postoperative acute kidney injury in patients undergoing cardiac surgeries. Interact Cardiovasc Thorac Surg.

