# Supplementary material for: Peri-operative fluid strategy and post-operative acute kidney injury in cardiac surgery patients: any role for pre-operative statin therapy?
Source: Crit Care. 2015 Dec 29;19:453. doi: 10.1186/s13054-015-1174-4 (PMC4752014; doi:10.1186/s13054-015-1174-4)
Supplement: Related texts

# Reply: Authors’ response

**Authors:** Ji-Yeon Kim, Eun-Ho Lee

We thank Professor Honore and colleagues for their interest in our article [1] and their comment. Statin has been widely used in patients with cardiovascular disorders. We agree that the patients in the control group were less likely to be receiving statin before the surgery, which could have affected our results. However, although some clinical studies have suggested a reno-protective effect of statin in patients undergoing cardiac surgery [6, 7], there is a lack of evidence to support the beneficial effect of pre-operative statin on renal function after cardiac surgery. Previous studies have reported that pre-operative statin therapy is not associated with a reduced incidence of post-operative AKI [8], including after cardiac surgery [9]. Nevertheless, we tested the impact of pre-operative statin treatment on our results. Although pre-operative statin treatment was not associated with post-operative AKI (odds ratio 0.95 (95 % confidence interval 0.65–1.39), *p* = 0.78) in a univariate analysis, we forced the variable into the final model and there was still a significant association between the renal protective fluid management strategy and post-operative AKI (odds ratio 0.23 (95 % confidence interval 0.14–0.36), *p* <0.001). We can therefore definitely conclude that statins had no significant role to play in our observations.
